# Managing collaboration between Traditional and Western health practitioners in mental health care, Limpopo province, South Africa

**DOI:** 10.4102/hsag.v30i0.2905

**Published:** 2025-07-22

**Authors:** Nare J. Masola, Mpho Maotoana

**Affiliations:** 1 Department of Psychology, School of Medicine, Sefako Makgato Health Sciences University, Pretoria, South Africa; 2 Department of Psychology, Faculty of Humanity, University of Limpopo, Polokwane, South Africa

**Keywords:** collaboration, mental illness, well-being, traditional health care systems, management

## Abstract

**Background:**

A significant proportion of South Africa’s black population seeks medical attention from both the traditional and Western health care systems. Traditional health practitioners (THPs) are consulted by many black Africans for the treatment of culturally bounded health problems. However, there is a limited understanding of how to manage the coexistence of traditional and Western health care systems in South Africa, despite government efforts to recognise THPs.

**Aim:**

This study aimed to understand the traditional health care providers’ perspectives on what could be done to manage cooperation among the health systems of Western health practitioners and THPs.

**Setting:**

This study was conducted in six rural villages in Mogalakwena Local Municipality, Waterberg district, Limpopo province, South Africa.

**Methods:**

Qualitative approach was adopted, and exploratory descriptive design was used. Thirty-seven participants were selected through purposive and snowball sampling techniques. Semi-structured interviews were conducted, and data were analysed thematically.

**Results:**

Four key themes emerged: (1) the formation of an organisation and formal registration of traditional health providers, (2) mutual understanding, (3) recognition and equal status in collaboration and (4) spirituality in traditional healing practices.

**Conclusion:**

Participants noted that effective governance could improve collaboration between health systems.

**Contribution:**

Adding to the existing studies, this study may also be of utility to policymakers aiming to improve the integration framework and to psychology practitioners who work with patients navigating both traditional and Western mental health systems. The study aims to inform the Department of Health on implementing workable partnerships.

## Introduction

Mental illness is a global public health concern, contributing significantly to the burden of disease across all continents. Mental diseases were responsible for around 12% of the total number of disability and loss globally, as reported by the World Health Organization (WHO 2002). These conditions – ranging from depression and anxiety to substance use disorders – impose substantial psychological, social and economic strains on individuals, families and health care systems (Jacob & Coetzee 2018). Although modern psychiatric tools such as the Diagnostic and Statistical Manual of Mental Disorders, Fourth Edition (DSM-IV): It is a tool used to classify mental health disorders (APA, 2021), psychological assessment batteries and various biomedical treatments are employed across continents including Asia, Europe and Australia, their long-term effectiveness in many developing nations remains limited. Factors such as unaffordability, overburdened clinical personnel and inadequate mental health infrastructure have rendered these approaches less accessible to large segments of the population, particularly in Africa (Moshabela, Zuma & Gaede 2016).

In response to these systemic gaps, many individuals in African countries resort to locally available health care alternatives. Traditional health practitioners (THPs) have continued to play a pivotal role in the diagnosis and management of mental health conditions. Cultural beliefs in regions like South Africa and Zimbabwe often associate mental illness with supernatural forces such as witchcraft, spiritual disturbances and the transgression of cultural norms (Azango 2015; Edwards 2011). These culturally embedded understandings influence help-seeking behaviours, making THPs the first point of contact for many individuals experiencing mental distress (Schoonover et al. 2014; Zuma et al. 2016).

In South Africa, mental health challenges remain prevalent, with research indicating that approximately one in six citizens are likely to suffer from a common mental disorder, including anxiety, depression or substance abuse (Zuma et al. 2016). Rural populations are particularly vulnerable, with studies showing higher symptom prevalence among individuals consulting traditional or primary health care services. Despite the widespread utilisation of THPs, the formal health care system in South Africa has historically functioned in parallel to, rather than in conjunction with, traditional practices.

The WHO’s 2002 report on traditional medicine prompted a surge in studies advocating for the integration of traditional and Western health care systems (WHO 2002). These investigations revealed that many patients in rural settings often seek assistance from THPs before consulting Western health practitioners (WHPs), a pattern observed across various African nations (Makgahlela & Sodi 2017; Neba 2011; WHO 2022). In alignment with these findings, the South African government enacted the *Traditional Health Practitioners Act No. 22 of 2007*, which recognises THPs as legitimate service providers and seeks to formalise their role within the national health care framework.

Efforts to integrate these dual health care paradigms aim to harness the strengths of both systems to improve health care outcomes. While various collaborative models have shown promise (Boum et al. 2021; Moshabela et al. 2016), systemic barriers such as scepticism, lack of standardised guidelines and differing epistemological foundations continue to hinder full integration (Audet et al. 2017). Nonetheless, the increasing policy focus on collaboration underscores a broader recognition of the need for culturally congruent mental health care, particularly in underserved communities. Ultimately, understanding the cultural underpinnings of mental illness and exploring the potential for collaboration between THPs and WHPs are not only relevant but also essential for strengthening mental health services in South Africa.

### Collaborations between South Africa’s traditional and western health systems around mental health

The South African government has advanced legislation and policies that control and support indigenous knowledge systems after discussions with relevant stakeholders and governing structures (Nemutandani, Hendricks & Mulaudzi, 2016). One of the outcomes of this was that South Africa officially recognised African Traditional Medicine through the implementation of the South African Traditional Health Practitioners Council and the adoption of the National Policy on African Traditional Medicine, which were established under the *Traditional Health Practitioners Act No. 22 of 2007*, which provides a legal framework for the registration, training and regulation of THPs (Tilley 2021; Van Rensburg 2009). In addition, policies such as the Integrated Health Policy (2013) and the National Health Insurance Bill (2019) have acknowledged the role of traditional medicine in strengthening primary health care, aiming to create a more inclusive and cooperative health care system (WHO 2022).

### Collaborative relationships between traditional and western health practitioners in the context of mental health

The typical cultural milieu in which THPs operate is not necessarily transferable to a medical setting, particularly a psychiatric institution, and vice versa. Consequently, the reality of facilitating formal professional relationships between the two health care systems in the context of mental health has not always been an easy road to walk. For example, findings by Kahn and Kelly (2001) indicated that THPs believed that visiting the hospital, rather than working there full-time, would be more pragmatic. Another study revealed that some THPs believe that WHPs fail to approach them with the respect that they deserve (Maluleka & Ngoepe 2018; Mokalapa 2020).

Similarly, some WHPs have opposed the presence of THPs in hospital settings because they believe that traditional medical techniques are unorthodox, unregulated or supported by science. This scepticism is frequently brought on by divergent medical ideologies, concerns about patient safety and the lack of established procedures for incorporating conventional healing techniques into official hospital settings. Nevertheless, despite challenges such as medical philosophies, lack of standardised regulations and scepticism from both traditional and Western practitioners, systemic collaborations are possible. For example, a study conducted in KwaZulu-Natal province revealed that although these two health care systems were wary of one another for multiple reasons, it is possible to bridge the gaps so that a sense of mutual understanding and respect could be developed and nurtured between them (Nkosi & Sibiya 2018). Similar results have been observed in other studies (Green & Colucci 2020; Zuma et al. 2016).

A literature review revealed that in Limpopo province, there is a relative paucity of peer-reviewed material regarding mental health and THPs. For example, the material regarding collaborations between the two health care systems has also been shown to be limited (Mokalapa 2020). The study aimed to understand the traditional health care providers’ perspectives on what could be done to manage cooperation among the health systems of WHPs and THPs, as the National Mental Health Policy Framework and Strategic Plan 2013–2020 emphasised the need to address the dependency between THPs and WHPs. These goals served as the foundation for the purpose of this study:

1. To ascertain the views of THPs on how best to regulate collaboration between the traditional and WHPs.
2. To ascertain the views of THPs about the best governance framework that could promote cooperation between the two health practitioners.

### Theoretical framework: Structural model of collaboration

**FIGURE 1:**
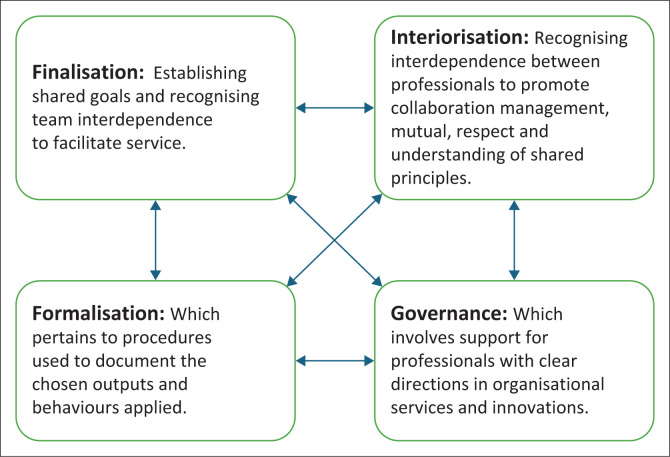
Theoretical framework: Structural model of collaboration.

This study employed D’Amour et al.’s (2008) structural model of collaboration. The model describes the collaboration between inter-professional and inter-organisational health organisations used in primary health care settings. The structural model of collaboration comprises four components that are described as follows (see Figure 1):

a) *Finalisation*, which argues that for successful collaborations, there should be shared goals, and that the recognition of team member interdependence can facilitate service provision (Chung et al. 2012); In line with this factor, the researchers explored what THPs perceive as shared goals between themselves and trained WHPs.

b) *Interiorisation*, which discusses how the recognition of interdependence between professionals can promote the management of collaborations and build mutual respect and understanding of shared principles and confidence (Chung et al. 2012). For this study, the researchers explored what THPs regard as important ways to manage collaboration to create a sense of trust between them and WHPs.

c) *Formalisation*, which pertains to procedures used to document the chosen outputs and behaviours applied.

d) *Governance*, which involves support for professionals with clear directions in organisational services and innovations (Chung et al. 2012). In this study, the researchers explored how THPs perceive as ideal structures for governance in both health systems.

## Research methods and design

The study adopted the constructivist paradigm, which acknowledges the socially constructed nature of knowledge and understanding, and the qualitative research approach, which is defined by Tenny et al. (2022) as a type of research method that seeks to explore and provide deeper insights into real-life problems.

### Research methodology

This study adopted a qualitative research approach. Qualitative research is suited for studies that aim to explore and understand how individuals and groups make meaning of social phenomena (Bricki & Green 2007). In the context of this study, the qualitative methods were appropriate for exploring how THPs in the Waterberg district municipality perceive as ways to manage collaboration between THPs and WHPs in the treatment of mental illnesses. This research strategy was suitable for achieving the goal of the study as it allowed the researchers to gather data from reliable sources and to provide the necessary analytic data.

### Research design

A descriptive exploratory design was used. This design is particularly valuable in the context where limit exists, enabling the researcher to describe the current practices and explore new insights into a relatively understudies area (AlYahmady & Saleh 2013). Given the lack of existing studies on managing collaboration between THPs and WHPs mental health care in the Waterberg district, this design provided the flexibility and depth necessary to capture nuanced understanding from perceptions of the THPs.

### Setting of the study

**FIGURE 2:**
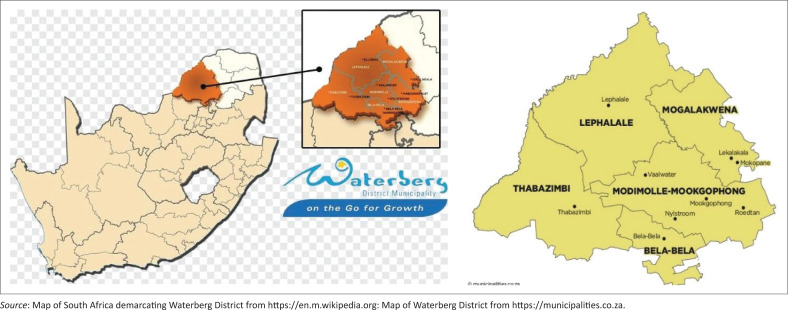
Map of South Africa demarcating Waterberg District and its local municipalities.

The study was conducted in six rural villages of Moshate, Mapela, Mozombane, Ga-Madiba, Masodi and Sekgakgapeng, which are located in Mogalakwena Local Municipality, Waterberg district, Limpopo province, South Africa. Mogalakwena Local Municipality covers a total area of 6156 km^2^ and includes villages, business services and mines. In all, 42 THPs are formally registered health care practitioners, specialising in a variety of health problems, including mental health challenges (Public Affairs Research Institute [PARI] 2017; Semenya & Potgieter 2014). The six villages differ in population size; linguistic composition, where Northern Sotho (Sepedi) is the dominant ethnic group; and access to health care services, where Sekgakgapeng, Ga-Madiba and Mapela have access to local clinic services but have limited access to specialised mental health services. Moshate, Masodi and Mozombane have no access to local clinic services, forcing residents to travel long distances for medical and mental care. Waterberg district and its local municipalities are shown in Figure 2.

### Sampling size and technique

A total of 37 THP experts, aged between 21 and 89 years, who specialise in mental illness and reside in six villages in Mogalakwena Local Municipality in Waterberg district were selected to participate in the study.

The selection process used a combination of purposive and snowballing sampling techniques. Purposive sampling involves the choice of participants in terms of predetermined standards, such as strong proof that they are a representation of the target community (Korstjens & Moser 2018); in this case, THPs with expertise in treating mental health conditions were chosen. The snowball sampling method is a non-probability technique that involves recruiting one participant and asking if they refer or suggest a second appropriate participant, who then suggests the name of a third and so forth (Korstjens & Moser 2018). The researchers followed these sampling techniques through two steps: firstly, the identification was based on pre-defined criteria, for example, because of good evidence that participants are well-known in the area for dealing with mental illness. Secondly, selected THPs were asked to recommend one or more THPs who deal with mental illness and may be interested in participating in the study. Participants were recruited through word of mouth.

### Data collection

The study employed semi-structured interviews with a predetermined set of open-ended questions to explore THPs’ perceptions of managing collaboration with WHPs. This method provided flexibility, allowing researchers to probe further into key areas while capturing participants’ in-depth insights. To ensure comfort and ease of expression, all interviews were conducted in Northern Sotho, known as Sepedi, the language that is most used in the areas. Interviews took place in a location chosen by participants. For accuracy, all interviews were audio-recorded, transcribed and back-translated. An independent, Northern Sotho-speaking researcher first translated the transcripts into English, and a third-party specialist reviewed them to ensure reliability. The study’s purpose and ethical consideration were clearly explained to all participants before data collection.

### Data analysis

Thematic analysis, following Braun and Clarke’s (2022) framework, was utilised to systematically identify, analyse and interpret patterns within the data collected through semi-structured interviews. The process began with an initial review of the transcribed interviews, where preliminary codes were assigned to recurring ideas in a reflexive manner. These codes were refined iteratively to ensure consistency and accuracy. Throughout the analysis, the researchers systematically reviewed and reassessed the data, applying clearly defined inclusion and exclusion criteria to maintain coherence in coding.

As the coding process progressed, relationships between codes were examined to identify emerging patterns. Codes that reflect similar concepts were grouped into broader themes, representing the core findings of the study (see Table 1). The iterative nature of the analysis allowed for continuous refinement, ensuring that the final themes accurately captured the depth and complexity of participants’ perspectives.

**TABLE 1:** Distribution of demographics (*N* = 37).

Categories	Frequency	Percentage (%)
**Age group (Years)**		
< 25	1	2.7
25–34	4	10.8
35–44	6	16.2
45–54	8	21.6
55–64	6	16.2
65–74	6	16.2
> 75	6	16.2
**Gender**		
Female	25	67.6
Male	12	32.4
**Location**		
Ga-Madiba	5	13.5
Mapela	6	16.2
Masodi	9	24.3
Moshate	7	18.9
Mozombane	4	10.8
Sekgakgapeng	6	16.2
**Experience (Years)**		
< 10	9	24.3
10–19	12	32.4
20–29	8	21.6
30–39	6	16.2
40–49	1	2.7
50–59	1	2.7
**Education level**		
Primary	8	21.6
High school	8	21.6
None	14	37.8
NSC	4	10.8
Tertiary	3	8.1
**Total**	37	100.0

NSC, national senior certificate.

### Trustworthiness and reflexivity

To ensure the credibility of this study, the researchers conducted number checking by seeking feedback from participants on the data, thereby verifying the accuracy and trustworthiness of the findings. To promote transferability, the researchers provided a detailed description of the sampling strategy and study context, and they discussed how the findings relate to existing literature from different settings to support the broader applicability of the results. For dependability, the team leader examined the data, findings and interpretations to ensure they were grounded in the evidence collected.

In terms of confirmability, reflexivity played a central role. The researchers acknowledged their positions as active contributors to the research process, aligning with Patnaik (2013), who emphasised that reliability and reflexive engagement enhance the reliability and legitimacy of qualitative findings. Methodological reflexivity and epistemological reflexivity were practised to monitor the influence of the researchers’ own beliefs and assumptions and to ensure that research decisions were transparent and justified. The team was also mindful of power dynamics between researchers and participants, promoting respectful and ethical engagement throughout the study.

### Ethical considerations

Ethical approval was obtained from the ethics committee of the University of Limpopo (TREC/204/2019/IR). Upon gaining approval, permission to gain access to the community of THPs was obtained from the Mogalakwena Traditional Association. Consent regarding voluntary participation, anonymity and confidentiality, and the recording of the discussions were obtained from participants during the data collection phase of the study.

## Results

Table 1 shows the demographic and geographic profile of the participants. The total number of the participants included in the study was 37 of which 25 were females and 12 were males, resulting in a sex ratio of 2.1:1. The total age of participants ranged from 21 to 89 years, with the mean age being approximately 55.4 years (standard deviation [s.d.] = 22.8). The participants come from different villages in Mogalakwena municipality, with the majority coming from Masodi and the least from Mozombane. The majority of THPs (32.4%) have 10–19 years of experience, with a notable portion also having 20–29 years (21.6%) and 30–39 years (16.2%). Very few (2.7%) have over 40 years of experience. While 37.8% of THPs have no formal education, 43.8% have only primary or higher education, and only 18.9% have NSC (National Senior Certificate) or tertiary education.

### Emergent themes

Emergent themes show four core themes with two sub-themes each that emerged from the findings and were used to structure the analysis and discussion of findings. The emergent themes are reflected in Table 2.

**TABLE 2:** Themes and sub-themes used for presenting the findings.

Themes	Sub-themes
1. Formation of an organisational and formal registration of THPs	1.1 Need for formal recognition 1.2 Regular meetings
2. Mutual understanding between traditional and western health practitioners	2.1 Intercultural learning 2.2 Shared knowledge
3. Recognition and equal status in collaboration	3.1 Formal oversight 3.2 Equal recognition
4. Spirituality in traditional healing practices	4.1 Ancestor guidance 4.2 Protection of traditional methods

THP, traditional health practitioners.

#### Formation of an organisation and formal registration of traditional health practitioners

Traditional health practitioners emphasised the need for formal recognition and representation to facilitate collaborations with WHPs. Participants noted that forming an official organisation would strengthen their professional standing and improve engagement with WHPs:

‘We should just form an organisation as Traditional health Practitioners. You see now, I am alone, if I were to approach a Western doctor on my own, they would probably think am crazy. But if we formed an organisation, they would understand our needs as Traditional health Practitioners.’ (P15, 76 years, female)

A significant barrier identified by participants was a lack of formal registration, which limited their ability to work alongside WHPs. Some THPs suggested that proper registration would lead to greater recognition and credibility:

‘Like I said, we could form an organisation so we can go to them, or we could register. A lot of Traditional Health Practitioners are not registered and therefore are not well-known.’ (P14, 60 years, female)

Participants also highlighted the importance of regular meetings between THPs and WHPs to build mutual trust and facilitate dialogue. They believed that organised gatherings could reduce misunderstandings and promote knowledge-sharing:

‘They could book a venue for us, and we would gather there as Traditional healers to win over Westerner doctors. It is not easy for Westerners to come to us in our respective places to come and enquire. But there, they could become interested.’ (P10, 81 years, male)

‘Our council for THPs and the Western medical council should meet and reach an agreement. Then, they should come back to us and tell us if we would be allowed to work in the hospitals and they could also come to us.’ (P19, 47 years, male)

Based on the above-stated findings, it is clear that the need for regular communication between the two health care systems cannot be overstated. The reduction of the gap between the two health systems will result in enhanced interaction and, subsequently, effective results and improvement in the health systems generally. This could further build confidence between the THPs and WHPs, improving the collaborations. The lack of formal registration of THPs is another obstacle identified by participants. Thus, increased formalisation of the THP sector could facilitate increased collaborations between the two health systems.

#### Mutual understanding between traditional and western health practitioners

Participants emphasised that collaboration between the two health care systems required mutual understanding and respect. They believed that the primary barrier was different beliefs about the causes of illnesses, which often resulted in scepticism from WHPs:

‘The biggest thing is reaching an agreement-we can’t work together without reaching an agreement. They know what they know, and I know what I know.’ (P13, 53 years, male)

‘… we can work together, we can agree. The big deal is just to understand each other.’ (P24, 35 years, female)

‘If only there was mutual understanding and respect – no oppression.’ (P15, 76 years, female)

Several THPs felt that WHPs viewed traditional healing as inferior, which created power imbalances. They stressed that both systems could complement, rather than, compete against, each other:

‘But I do not think that is doable, a lot of western health practitioners undermine traditional health practitioners. If they would allow us to show them that we have the ability …’ (P11, 83 years, female)

‘We should note that western practitioners do not like working with us because they think we are after their jobs, which is not true.’ (P20, 50 years, male)

‘If someone says “am a tiger, am a leopard” how will they work together? …’ (P30, 67 years, male)

‘It can bring good things because when we work together with Western health Practitioners, we make sure that people get life. You find that there is something I don’t know, but they know it, we mix, and it becomes something, and people heal.’ (P12, 42 years, female)

Communication was identified as a key enabler of collaborations. Participants suggested that structured dialogues and knowledge-sharing sessions would improve the interactions between THPs and WHPs:

‘The biggest thing is communication. If we could participant with the Western health Practitioners and have a talk on how we do things and unite, we would conquer over these diseases that the Western health Practitioners cannot heal but treatable in the traditional healing approach.’ (P24, 35 years, female)

‘We must communicate with qualified practitioners … there are going to be a lot of changes.’ (P14, 60 years, female)

Based on the above-stated extracts, traditional health care providers encourage mutual understanding when it comes to patient care. The view of the participants is that a dialogue between the two health care systems would be a practical mechanism to improve collaborations between individual practitioners, as well as the overall health care system.

#### Recognition and equal status in collaboration

Traditional health practitioner highlighted the importance of being equally recognised and respected in collaborative settings. They expressed the need for fair treatment and equal status, arguing that traditional healing should be acknowledged at the same level as Western medicine:

‘Because we are under oppression, they must give us equal rules and recognition without favour besides them making you feel small. I shouldn’t be oppressed because I am using bones.’ (P08, 43 years, male)

‘If our leaders would fight for us, for our names to be entered into the database along our specialties it would help.’ (P13, 53 years, male)

Participants also mentioned the importance of aligning such recognition with both national legal frameworks and traditional leadership structures, as outlined in the *Traditional Health Practitioners Act No. 22 of 2007*:

‘I think we should be ruled by our leaders’ rules and be ruled by the rules of our country. The police should be involved in our affairs, tribal council should also be involved because we are led by kings.’ (P16, 77 years, female)

Based on the findings, the participants believed that the recognition that is governed in line with the *Traditional Health Practitioners Act No.22 of 2007*, which aims to regulate THPs and integrate them into the broader health care system may facilitate the smooth management of the two health systems.

#### Spirituality in traditional healing practices

Simultaneously, spirituality emerged as a core and non-negotiable aspect of traditional healing. Some THPs described their practices as being directly guided by ancestral instructions, which they believed should not be compromised, altered or disclosed in ways that might dilute their sacredness during collaboration:

‘Our spirituality governs us. Our ancestors are the ones ruling over us …’ (P18, 76 years, female)

‘We ask our ancestors for permission; we do not govern ourselves. Our ancestors do not allow us to share with anyone how we do things.’ (P11, 83 years, female)

‘Our spiritual laws are sacred we can’t take our spirituality and start practicing them in Western sector. We should maintain our traditional ways, or we would be contaminating them. We want them raw as they are, uncontaminated.’ (P20, 50 years, male)

Based on the preceding extracts, the participants emphasise that the government should acknowledge the spiritual foundations of traditional medicine and preserve its unique practices. In that way, the systems will be managed effectively.

## Discussion

This study explored the perceptions of THPs regarding collaborations with WHPs within the South African health care system. The findings revealed that THPs perceive themselves as sharing a common goal with WHPs to save lives, a sentiment supported by Mokalapa (2020). This aligns with participants’ views that both systems serve the community and should complement one another rather than compete. Traditional health practitioners in the study recognised that working together could expand the range of services available to patients, especially those who consult both systems. This is consistent with Van-Niekerk et al. (2014), who found that cooperation between THPs and WHPs could lead to improved disease management and diversified treatment approaches. Some participants expressed that such collaboration could help integrate traditional healing perspectives into mainstream treatment, while also enhancing scientific innovations through the inclusion of traditional knowledge. These views support Habtom’s (2018) suggestion that collaborative efforts may lead to the standardisation of traditional medicines and facilitate mutual learning between systems. However, as Moshabela et al. (2016) noted and as echoed in this study, the distinct operational methods of the two systems, spiritual versus biomedical, often make shared objectives difficult.

Working together was viewed by participants as particularly important for the field of mental health. The study found that many THPs believe mental illnesses often have spiritual causes and are best addressed through culturally grounded approaches. Some participants expressed that collaboration could allow patients to benefit from both traditional and regulated biomedical treatments. This reflects findings from Mokalapa (2020), which suggested that WHPs recognised the benefits of collaboration for patients and the broader health care system. Participants also mentioned that cooperation could improve treatment experiences in mental health facilities by allowing for referrals across systems, reducing duplication of care and respecting patient preferences. However, consistent with Moshabela et al. (2016), the study also revealed that WHPs often view traditional healing as unregulated and scientifically unfounded, contributing to a lack of trust.

Participants in this study reported mixed experiences with WHPs: while some described respectful and open-minded professionals, others shared experiences of rejection, marginalisation and being dismissed as illegitimate. This finding aligns with Mokalapa (2020), who noted that some WHPs were receptive to collaboration while others viewed it as impractical. Participants also raised concerns about how delays in seeking hospital care – often blamed on THPs – were misunderstood and used to justify exclusion. In this context, collaboration was seen as a potential solution to bridge service gaps and improve patient referral pathways. As Van-Niekerk et al. (2014) argued, allowing patients to choose their treatment path empowers them and supports pluralistic health care. Yet, this study confirmed that a small number of WHPs viewed the partnership as unlikely to change outcomes, further highlighting the need for mutual education and formal mechanisms to support collaboration.

To formally register and represent THPs, participants indicated a strong demand for the establishment of a registered body or organisation. Traditional health practitioners lack the credibility and voice necessary to interact with WHPs and feel alienated from the health care system in the absence of official registration or an organisational structure. This result also confirms other studies (Van Rooyen et al. 2015), which found that formal frameworks aid in guaranteeing accountability and acknowledgement in integrated care models. It was recommended that the South African government and all other interested parties collaborate to record the incorporation of THPs into the health care system, according to a 2018 study conducted by Habtom, maintaining records of traditional medicine is crucial as it raises its standing within the public health systems. The framework that allows for continuous communication between the governing bodies is optimal. The importance of open communication and collaborative knowledge sharing in bridging cultural and epistemological divides was stressed by the participants. This corroborates the conclusions of Patel (2011), who asserted that cross-system partnerships must be founded on intercultural conversation and trust. The research indicates that collaborations may only thrive when both systems recognise the distinct contributions each offers to health care, especially in addressing culturally specific ailments.

The study found several important elements that might help THPs and WHPs to work together successfully. The establishment of trial periods for new policies before their formal acceptance is one crucial suggestion. This strategy would foster trust and understanding between the two systems and enable the assessment of policy efficacy. Enhancing both groups’ management and communication abilities was also highlighted as an essential first step. According to the study, which supports the findings of Nemutandani et al. (2016), focused training programmes that emphasise networking, communication, cooperation and associated competences could significantly enhance cooperative efforts.

The study also emphasised how crucial cross-education between the two systems is. Mutual respect and trust could be promoted by giving WHPs and THPs thorough information about one another’s areas of expertise, therapeutic approaches and ethical frameworks. Reducing stress in the health care system and enhancing working relationships depend on developing this trust. This kind of cooperation could increase access to mental health care, reduce hospital pressures and provide patients with more options for treatment. In addition, it would guarantee that patients have access to appropriately licensed traditional health professionals, improving the general standard and inclusiveness of health care delivery.

The research identified two factors that might enhance and inhibit collaboration. The system used for referral was identified as an obstacle. Western health practitioners refer patients only to one another and don’t include the THPs, and this exclusion makes WHPs not to recognise THPs as member of the system but see them as autonomous entities. In a study conducted by Nkosi and Sibiya (2018), it was noted that the formal referral system between THPs and WTPs was initiated but the National Department of Health (NDoH) was unwilling to approve it. In addition, there are no data to show the outcomes of the conventional drugs, because of a lack of reports, and therefore, WHPs hesitate to collaborate with them (Mokalapa 2020).

### Limitations of the study

The study’s scope is limited to the perception of THPs in the Waterberg district. Few THPs with NSC or higher education level understand the concept of collaboration better. The questioners are conducted in Northern Sotho, and therefore, the translation of the data from the transcription as documented might temper with the true nature of the expression of THPs opinion on the matter.

### Contributions and recommendations

The study contributes to the Department of Health by structuring and implementing collaboration, despite the restrictions and the fact the study only covered one district in Limpopo province. It also contributes to the small body of extant literature on teamwork. By giving policymakers insights into and understanding of THP ideas on collaboration, it will further aid in the formulation of collaboration policies. The study will demonstrate the necessity and significance of updating the current policies to include comprehensive collaboration guidelines and creating a formal referral system that would oversee the cooperation.

Further research on collaboration between the two practitioners is necessary, particularly in Limpopo province and provinces in South Africa. To support effective integration between THPs and WHPs, Council should offer formal training programmes from both systems. In addition, the government should consider incorporating THPs into mental health care facilities within the Western health care sector, thereby promoting a more inclusive approach to mental treatment. Policymakers have a critical role to play in establishing and documenting official guidelines for collaboration and referral systems.

## Conclusion

In Waterberg district, the collaborative environment has a positive outcome. Traditional health practitioners shared positive views regarding the idea of collaboration. The concept of teamwork was seen favourable by THPs. According to the study, THPs and WHPs do have some similar objectives. Although the two systems are unaware of one another, they both aim to improve mental disease management and treatment. They were unaware of the laws that encouraged the partnership. The government failed to ensure both practitioners follow the guidelines that can ensure collaboration is practised. The THPs are willing to exchange knowledge with WHPs. On the contrary, the collaboration will benefit the practitioners by lowering the waiting lists and the workload. In addition, the health care users provided them with a variety of services that they find meaningful and a choice of treatment options. The necessity for a formal referral that enables WHPs to refer patients to THPs was identified. The THPs also propose that for them to be formally recognised, their names and specialties should be entered into an official database.
